# Auxin analysis using laser microdissected plant tissues sections

**DOI:** 10.1186/s12870-018-1352-z

**Published:** 2018-06-25

**Authors:** Luz G. Muñoz-Sanhueza, YeonKyeong Lee, Molly Tillmann, Jerry D. Cohen, Anne Kathrine Hvoslef-Eide

**Affiliations:** 1 Department of Plant Sciences (IPV), Faculty of Biosciences, Norwegian University of Life Sciences, Norway Campus Ås, Universitetstunet 3, 1430 Ås, Norway; 2 Department of Horticultural Sciences, Microbial and Plant Genomics Institute, University of Minnesota, 305 Alderman Hall, 1970 Folwell Avenue, Saint Paul, MN 55108 USA

**Keywords:** Auxin quantification, Isotope dilution analysis, Laser microdissection microscope, GC-MS/MS quantification, Plant sample preparation, Minute samples, Freeze drying, Lyophilisation, Cryosectioning

## Abstract

**Background:**

Quantitative measurement of actual auxin levels in plant tissue is complimentary to molecular methods measuring the expression of auxin related genes. Current analytical methods to quantify auxin have pushed the limit of detection to where auxin can be routinely quantified at the pictogram (pg) level, reducing the amount of tissue needed to perform these kinds of studies to amounts never imagined a few years ago. In parallel, the development of technologies like laser microdissection microscopy (LMD) has allowed specific cells to be harvested from discrete tissues without including adjacent cells. This method has gained popularity in recent years, especially for enabling a higher degree of spatial resolution in transcriptome profiling. As with other quantitative measurements, including hormone quantifications, sampling using traditional LMD is still challenging because sample preparation clearly compromises the preservation of analytes. Thus, we have developed and validated a sample preparation protocol combining cryosectioning, freeze-drying, and capturing with a laser microdissection microscope to provide high-quality and well-preserved plant materials suitable for ultrasensitive, spatially-resolved auxin quantification.

**Results:**

We developed a new method to provide discrete plant tissues for indole-3-acetic acid (IAA) quantification while preserving the plant tissue in the best possible condition to prevent auxin degradation. The method combines the use of cryosectioning, freeze-drying and LMD. The protocol may also be used for other applications that require small molecule analysis with high tissue-specificity where degradation of biological compounds may be an issue. It was possible to collect the equivalent to 15 mg of very specific tissue in approximately 4 h using LMD.

**Conclusions:**

We have shown, by proof of concept, that freeze dried cryosections of plant tissue were suitable for LMD harvest and quantification of the phytohormone auxin using GC-MS/MS. We expect that the ability to resolve auxin levels with both spatial- and temporal resolution with high accuracy will enable experiments on complex processes, which will increase our knowledge of the many roles of auxins (and, in time, other phytohormones) in plant development.

## Background

With the ability to analyse very low hormone levels comes the desire to be able to sample very specific plant tissues in order to uncover the precise hormonal changes regulating development. Phytohormones are often active in very specific tissues or cell layers within a tissue, and the ability to distinguish one cell type from another can be revolutionary in the understanding of these responses. Auxins can serve as an example of the more delicate phytohormones, and are involved in a plethora of different plant growth and developmental responses, including lateral and adventitious root formation, cell expansion, apical dominance, gravitropism, and abscission, among others. The effect of a mobile auxin signal was first described in the latter half of the nineteenth century by Ciesielski [1] on root responses to gravity, but the chemical identity of the signalling compound was not discovered until the first half of the twentieth century that identified indole-3-acetic acid [2].

The physiological response to auxin is highly dependent on local concentrations. Thus, biosynthesis, degradation, and transport of auxin play central roles in maintaining what appears to be a delicate homeostatic equilibrium among auxin precursors, free hormone, and conjugates.

As we discussed previously [3], spatial-temporal resolution of auxin activity has been visualized using various reporter systems. These methods are based on constructs using synthetic promoters such as DR5 [4] or co-receptors such as the fusion protein DII-VENUS [5]. They have been developed, and are particularly useful for reference plant species with standardized transformation protocols. Although these techniques have important utility for understanding sites of auxin activity they do not quantitatively measure auxin levels and, as Liao et al. [6] emphasized in their report, “It is important to note that neither reporter shows a linear response to auxin concentrations or treatment duration, and hence these cannot be used to infer actual auxin levels.” Nevertheless, these methods complement the absolute quantification procedure described here for measurements in specific tissues.

The capacity to detect and quantify auxin metabolites in plant tissues has been a major driving force in plant biology since the first bioassays were developed in the 1920s and 1930s [3]. Auxins are present in very low concentrations in plant tissues, typically in the range of 5–50 ng/g fresh weight [7]. We have found that poinsettia flower buds have, in agreement with these general ranges, IAA concentrations in the range of 1.2 to 55 ng/g FW (Hvoslef-Eide, et al., manuscript in prep). Early studies required kilogram amounts of plant materials, as well as days to months of effort, for a single measurement [3]. To overcome these limitations, improved auxin purification methods and the utilization of increasingly more sensitive instruments have been developed. Since its initial application for quantification of auxins, isotope dilution [3] has played an important role in auxin quantification by mass spectrometry. It has allowed the application of modern sample manipulation and the utilization of constantly improving instrumentation, to such an extent that the procedures have reached impressive limits, especially regarding to the amount of plant material needed for an analysis [8]. These highly sensitive physical methods for analysis have the potential to provide insight into the role of auxin in the plant’s physiology because they open up the possibility to measure auxin distribution with high spatial resolution [9].

Laser microdissection microscopy (LMD) allows specific cells to be harvested from complex tissues, even to the level of the single cell, providing a starting point for downstream analyses including quantitative real-time polymerase chain reaction (PCR), microarray, DNA genotyping, RNA transcript profiling, generation of cDNA libraries, etc. This technology has been available since 1996 [10], but the first application in a plant study was not until 2002 [11]. LMD has been primarily used in combination with RNA isolation and gene expression studies [12, 13], which can be accomplished on paraffin-embedded tissue. LMD applications for analysis of small molecule metabolites, especially phytohormones, present additional challenges due to the intrinsic low concentration of these molecules, their solubility in embedding matrix materials and water, and the possibility of compound degradation, especially under ambient temperatures. These characteristics make traditional LMD protocols inappropriate for auxin sample preparation because use of solvents and fixatives during the dehydration and fixation processes would solubilize and/or result in degradation of the hormone in situ. In order to maintain the original phytohormone content of the tissues, cryosectioning followed by freeze-drying has been applied, since freeze-drying does not alter the hormone content [14]. Cryosectioning has more typically been applied to animal tissues rather than plant tissues because the presence of vacuoles and cell walls in plants often make it difficult to preserve the integrity of cell structures [15, 16]. However, an increase in thickness of the cryosections improves results with such tissues. Here, we report an efficient sample preparation method that combines three steps: (1) cryosectioning of the plant tissue, (2) freeze-drying of the cryosections, and (3) harvesting the cells using LMD. This protocol describes collection of plant materials for the subsequent auxin extraction and quantification using the GC-MS/MS and isotope dilution [8]. Due to the lack of protocols that combine the use of LMD and auxin quantification, and since an important role for auxin in plant development is its function as a positional signal [9], this work has potential for leading to a significant improvement in the quality of information provided by hormone analysis.

## Methods

### Plant materials

Cuttings with at least two leaves approximately 5 cm long were harvested from poinsettia (*Euphorbia pulcherrima,* Willd. ex Klotzsch) ‘Millenium.’ Mother plants and cuttings were grown under long day conditions (16 h light, 22 °C day and 20 °C night) and cuttings were kept in 70% relative humidity (RH) for four weeks. After the cuttings were rooted, they were transferred to 12 cm pots and kept under the same conditions for an additional two to three weeks. To induce flowering, the plantlets were transferred to short day conditions (10 h light, 20 °C) and RH 74%. The inflorescence of poinsettia is arranged with a main flower (first order flower), surrounded by three second order flowers, in turn surrounded by six third order flowers [17]. Six third order flower buds of identical development were used in this study.

### Abscission induction by flower bud decapitation

**Fig. 1:**
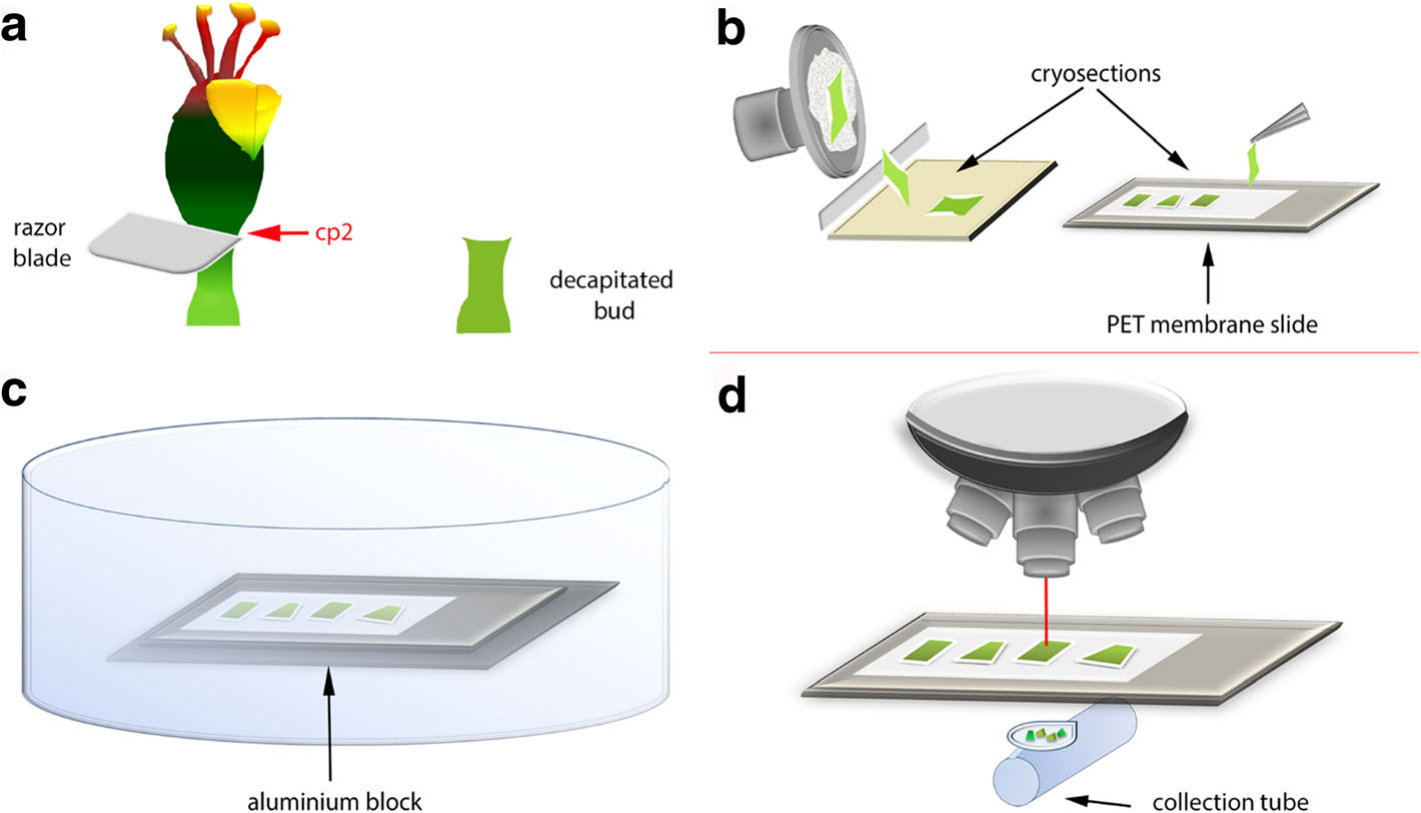
Summary of steps in sample preparation protocol for poinsettia. **a** Decapitation of flower bud. **b** Cryosectioning at 250 μm thickness and the placing the cryosections on PET membrane slides. **c** Freeze drying the PET membrane slides with cryosectioned samples. **d** Laser microdissection microscopy. Cp2, cutting point 2

When third order flower buds were fully developed, they were decapitated with a razor blade at cutting point 2 according to our previous publications (cp2; Fig. 1a). By doing this, the floral organs are removed but the remaining flower is kept intact [18, 19]. This induces formation of the abscission zone, which was visible after approximately four days (D4), and the bud abscised approximately seven days after induction (D7).

### Validation of the method with control samples

**Fig. 2:**
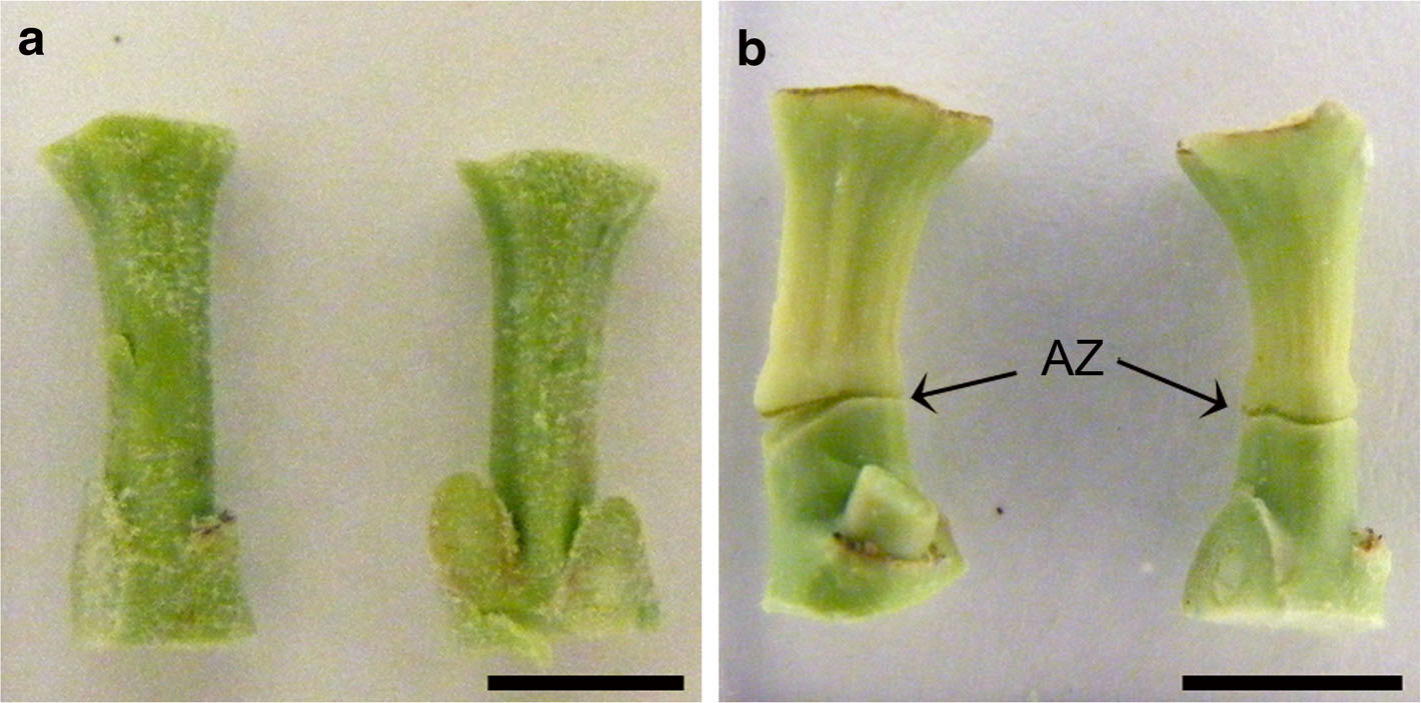
Decapitated poinsettia flower buds; **a** Day 0. **b** 6 days after decapitation. AZ, abscission zone. Arrows indicate abscission zone. Bars 5 mm

**Fig. 3:**
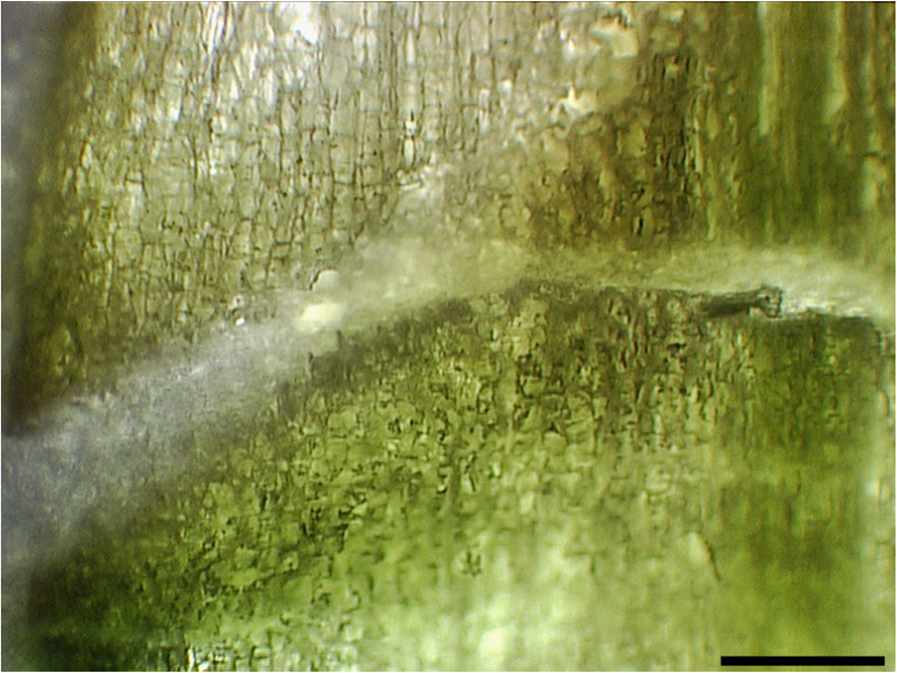
Longitudinal section of poinsettia flower bud at 6 days after decapitation (D6), abscission zone can be identified from its inverted cone shape. Bar, 310 μm

To validate the biological sampling method, we collected the area of interest within the abscission zone from the bud immediately after decapitation (D0) and then from the abscission zone six days after decapitation (Fig. 2). The abscission zone typically has a very irregular tri-dimensional cone shape, thus making it difficult to collect manually without inclusion of adjacent cell layers; this characteristic makes it an ideal candidate for the precision of LMD (Fig. 3). The results of this sampling were compared with simple cross sections of flower buds collected at the same stage in the abscission zone area. The difference between the experimental samples and the control samples was a comparison of freeze-dried tissue harvested with precision using the laser microdissection microscope in contrast to frozen cross sections that included unwanted cell types. We thus expected the results to fall within the same order of magnitude, but would not necessarily yield exactly the same values for the tissue IAA concentrations.

### Materials

- Tissue-Tek® OCT™ Compound and Cryomolds® (Sakura Finetek, Netherlands)
- Frame slides PET (polyethylene terephthalate)-membrane 1.4 μm, ref. n° 11,505,190 (Leica Microsystems, Germany)
- Tweezers 2A (A. Dumont & Fils, Switzerland)
- Custom-made aluminium blocks 3.0 cm × 8.5 cm × 0.4 cm
- RNAse free Microcentifuge tubes 0.6 ml
- Frame support Leica n°11,532,325 (Leica Microsystems, Germany)
- Accu-Edge® 4689 Low Profile Microtome Blades (Feather Japan)

### Equipment

- Cryostat Microtome HM560 (Microm, Germany)
- Freeze drier with top container (Heto Holten A/S, Allerød, Denmark)
- Laser microdissection microscope Leica 6000 (Leica Microsystems, Germany) with software V6.7.2.4295
- Laser Leica CTR 6500 (Leica Microsystems, Germany)

### Pre-sampling procedures

The abscission zone tissues from poinsettia flower buds in two different stages of the abscission zone progression were selected for this study, and specific cell layers corresponding to the abscission zones were harvested using LMD. The morphology of the abscission zone is very characteristic, making it easy to identify under a microscope. Three biological replicates were used in this study. An average of two flower buds per replicate were used for Day 0 and 2.8 flower buds for Day 6. Every replicate was subjected to: (1) the sample preparation procedure consisting of cryosectioning, freeze-drying, and LMD (Fig. 1), and (2) the final analytical procedure consisting of auxin extraction, derivatization and quantification using the protocol developed by Liu et al. [8]

This protocol includes five parts:

1. Longitudinal cryosectioning of flower buds using a cryostat
2. Freeze-drying cryosections generated from the previous step
3. LMD of freeze-dried cryosections in order to harvest specific tissue in the abscission zone
4. Method validation
5. Auxin extraction from microdissected tissues and quantification by GC-MS/MS

#### Tissue collection and cryosectioning

D0 buds were decapitated and collected immediately, and D6 buds were harvested six days after decapitation. For both time points, whole decapitated buds were harvested and placed in liquid nitrogen immediately after collection and stored at − 80 °C until cryosectioning. Cryosectioning was performed using a Cryostat Microtome HM560 (Microm, Germany) with an Accu-Edge® 4689 Low Profile Microtome Blade (Feather Japan). The temperature of the blade and specimen (sample holder) were modified in the cryostat to − 16 °C and − 15 °C, respectively; optimal temperatures can vary from tissue to tissue depending on their macro structure. To find the optimal combination for every sample type, trial tests were necessary with test plant materials.

**Fig. 4:**
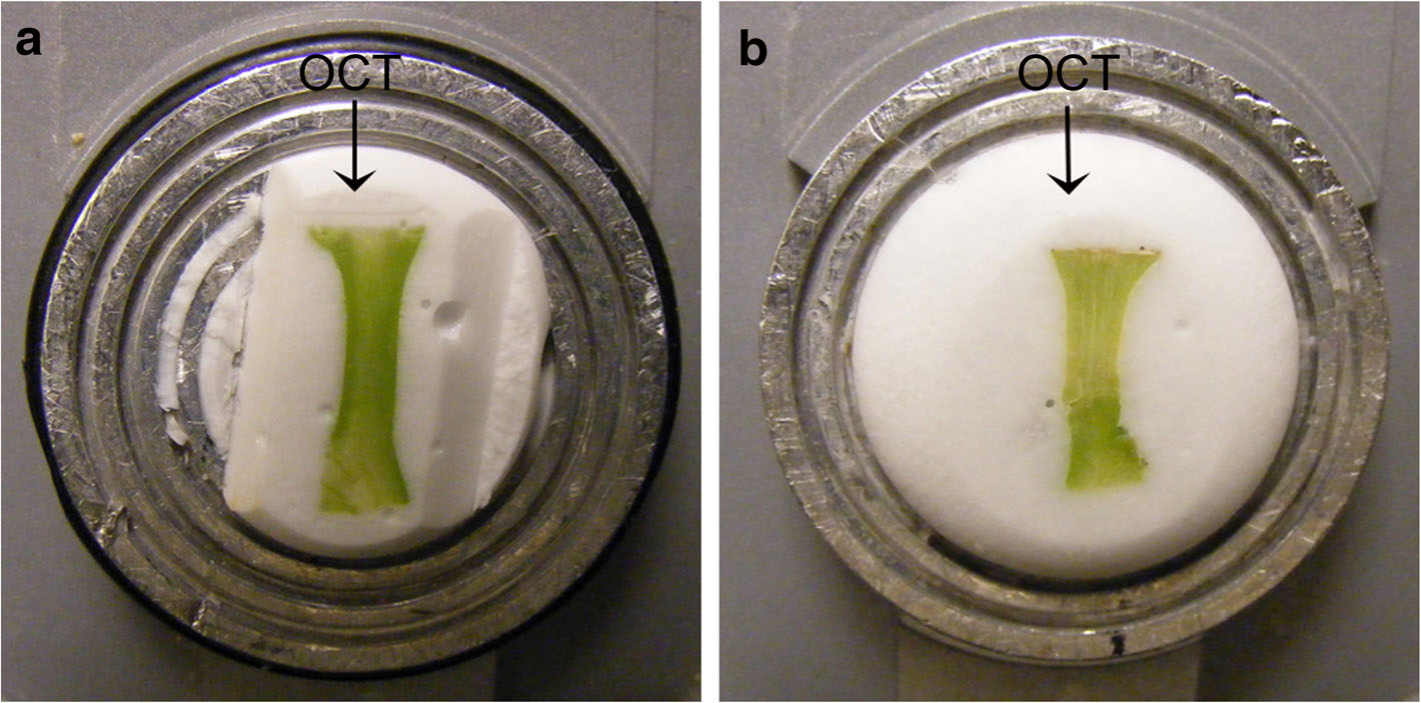
Sample holder with poinsettia buds in OCT compound ready for cryosection. **a** Bud at D0 (day of decapitation). **b** Bud at D6 (6 days after decapitation). OCT: optimum cutting temperature. Arrows indicate OCT compound

**Fig. 5:**
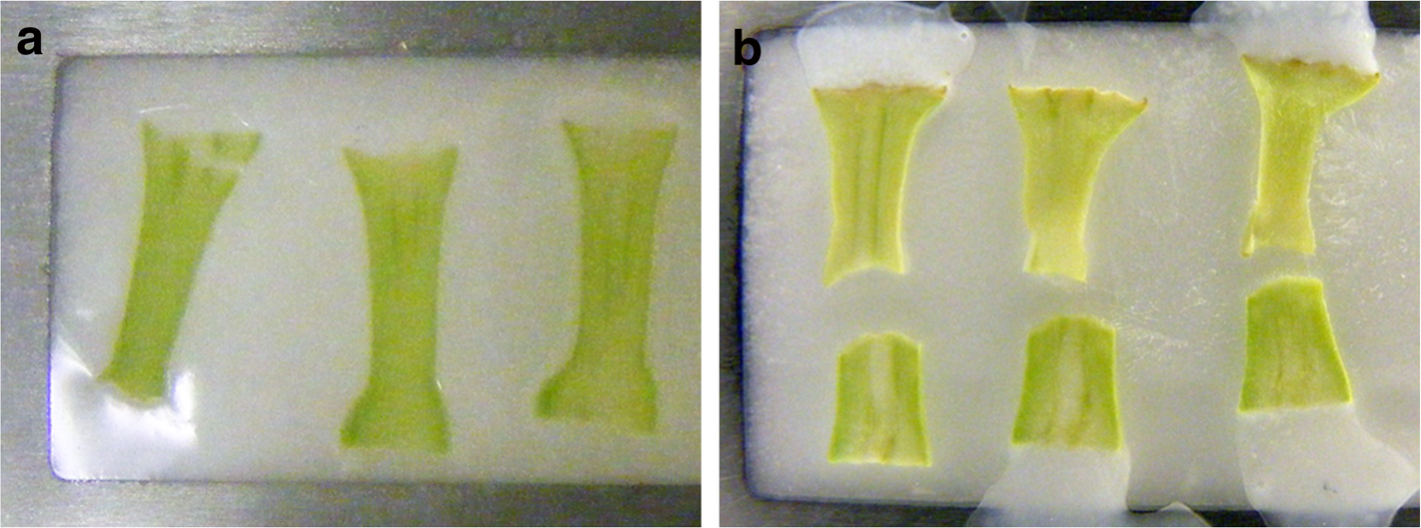
PET membrane slides with cryosections of poinsettia buds. **a** D0 (day of decapitation). **b** D6 (six days after decapitation)

The desired cryosection thickness can also differ depending on the goal of the study and the nature of the tissue. In this study, the thickness was typically about 250 μm, although acceptable results were obtained from sections ranging from 70 μm to 350 μm. The flower bud was placed on the sample holder using a drop of Optimal Cutting Temperature (OCT™ Tissue-Tek® Compound and Cryomolds®, Sakura Finetek, Netherlands) embedding medium to keep it fixed in the desired position (Fig. 4). Some extra OCT was added around the flower bud just before beginning the fast freeze process inside the cryostat chamber. Excess OCT can be removed from the edges of the tissue with a razor blade when needed. In order to prevent degradation of auxins during cryosectioning, straight tweezers with flat tips (Tweezers 2A A. Dumont & Fils, Switzerland) were used to manipulate the flower buds while the flower buds were kept on dry ice. Once the cryosections were transferred to the PET membrane slide using the cold tweezers, a Leica acrylic frame support (n°11,532,325, Leica Mycrosystems, Germany) was placed directly under the slice for several seconds to help the cryosections adhere to the slide surface. When placed correctly, the tissue and OCT will both spread evenly onto the membrane. This placement was done quickly to avoid exposing the cryosections to higher temperatures. Three to four cryosections were placed on each slide (Fig. 5). While OCT was useful in helping cryosections adhere to the slide membrane, it must be used with care to avoid contamination of the tissue through liquefying. Cryosections can stick to the membrane without OCT, but tissue edges may fold over during the freeze-drying process resulting in loss of the sample from the PET membrane slide. The PET membrane slides were kept in a controlled temperature chamber at − 19 °C in the cryostat or on dry ice after each cryosection.

#### Freeze drying

**Fig. 6:**
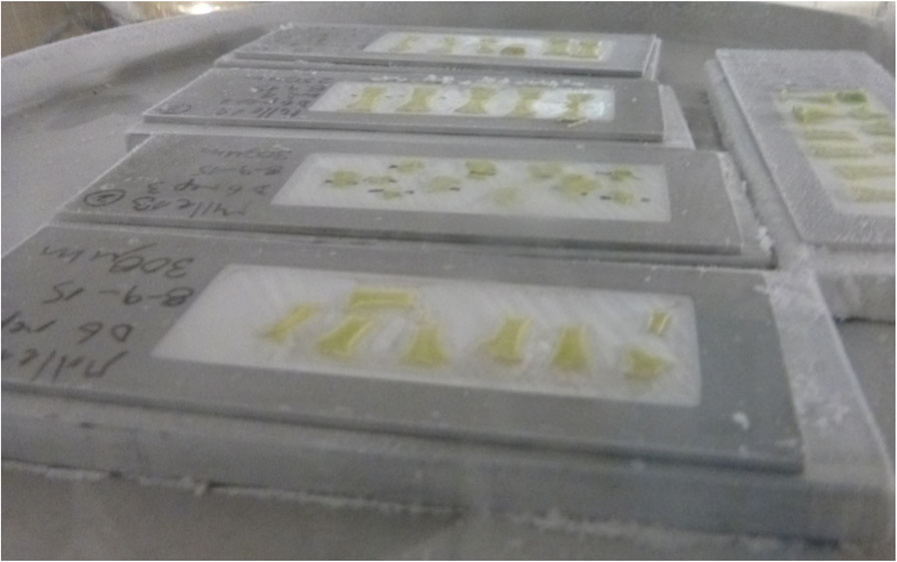
4 mm thickness aluminium blocks under the PET membrane slides during the freeze dry process

The PET membrane slides with the cryosections were placed on top of pre-frozen custom-made aluminium blocks of 4 mm thickness in order to maintain them in a frozen state (Fig. 6). The cryosections were freeze-dried in vacuo until the tissues were totally dried. The freeze-dried slides were stored at − 80 °C until microdissection. To test the tissue integrity and the feasibility of distinguishing desired structures after freeze-drying, different thicknesses of tissue sections were evaluated (70, 100, 200, 250 and 300 μm).

#### Microdissection

Microdissection was performed using a Leica 6000 laser microdissection microscope (Leica, Mycrosystems, Germany). The laser (CryLaS FTSS 355–50, Germany) was turned on at least 20 min in advance to allow it to warm up before starting the dissection. Laser parameters were set using Laser Microdissection software (V6.7.2.4295, Leica Mycrosystems, Germany) as follows: power 60, aperture 45, speed 20, and specimen balance 0. These parameters, optimized for the material we used in this study, can be adjusted in order to obtain the best conditions for every new tissue. The cutting position of the laser was calibrated using a new PET membrane slide or an area of membrane without any tissue. A clean microcentrifuge tube was placed in the collector device under the microscope stage and a slide with the cryosections was placed in the slide holder to start the dissection. The area of interest was selected and dissected, and the microdissected tissues were collected by gravity.

Microdissection was performed on only one slide at a time, and the remaining slides were kept cold in the dark (both important to prevent IAA degradation). All cryosections collected were 250 μm thick, and section volumes were calculated by multiplying the area by the thickness. Because the fragility and small size of the microdissections make them difficult to handle for weighing with a balance, fresh weight information can be more accurately calculated in uniform plant samples using volume to weight conversions determined from larger samples.

#### Validation samples

To validate the methodology, the flower buds providing the control material were frozen in liquid nitrogen and cross-sectioned with the cryostat around the area of the abscission zone. Three buds were used for each time point. Since the samples were frozen immediately after collection, the weight of each cross section was calculated by measuring the areas with the program ImageJ 1.49n (Rasband, W.S., ImageJ, U. S. National Institutes of Health, Bethesda, Maryland, USA, https://imagej.nih.gov/ij/, 1997–2016) and multiplying the result by 0.5 mm according to the thickness of the cross sections (500 μm). Reproducibility was evaluated in the control samples and the LMD samples by considering the average of the three measurements and calculating the standard error. Auxin quantification was performed in the same manner as the experimental material [8].

#### Auxin quantification

The protocol for auxin extraction [8] is optimally performed in 1.5 ml microcentrifuge tubes; thus, the tissue collected with LMD in a 0.6 ml microcentrifuge tube was transferred. Due to the small size of the cryosections and the potential presence of the slide membrane underneath them, electrostatic forces require that extra care must be taken. The protocol used in this study can be used to quantify auxin, as well as auxin biosynthetic precursors like tryptophan, indole, indole-3-pyruvic acid (IPyA) and indole-3-butyric acid (IBA). This protocol employs isotope dilution using [^13^C_6_] IAA as the internal standard [20] and requires only 2 to 20 mg of plant material. Approximately 340 pg of internal standard was added to every replicate in this study. The metabolite extract was derivatized with diazomethane [21] and analysed by selecting reaction monitoring (SRM) mode on a GC-MS/MS according to Liu et al. [8]. A small modification to the original protocol was made in the final resuspension step following derivatization, using 10 μL of ethyl acetate instead of 15 μL. Samples were analysed by GC-MS/MS immediately after preparation in this study, although when necessary, samples can be stored at − 80 °C.

## Results

We have described a reliable protocol for sample collection and auxin analysis using small amounts of discrete plant tissues. The protocol combines cryosectioning plant tissues, freeze-drying these cryosections, and laser microdissection for harvesting the specific cells or cell layers. IAA levels are then quantified in the collected plant material by GC-MS/MS with [^13^C_6_] IAA as an internal standard.

### Cryosectioning and freeze-drying

**Fig. 7:**
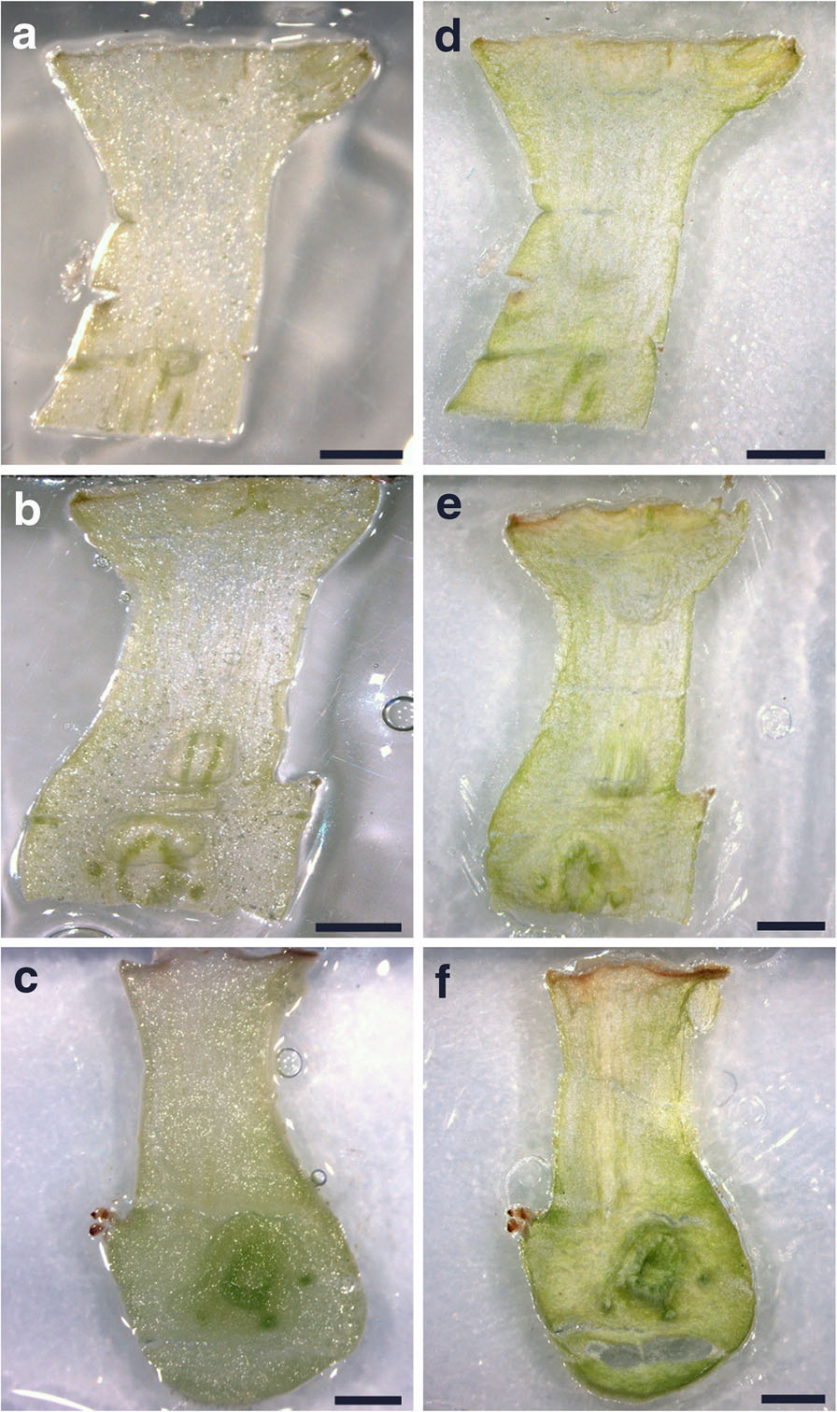
**a**, **b**, **c** Frozen cryosections of poinsettia buds at 70 μm, 100 μm and 200 μm respectively. **d**, **e**, **f** The same as in a, b and c but freeze dried cryosections. Bars, 1 mm. The preservation of the integrity is clear in all the cryosections

1. The integrity of all the cryosections from 70 μm to 200 μm thickness were well preserved after freeze-drying (Fig. 7).
2. The integrity of the cryosection at 250 μm thickness was well preserved after the freeze drying process and was the most suitable thickness for the bud tissue studied
3. For some studies, thicker cryosections (300 to 350 μm) may be desired. These thicknesses were also found to preserve the structure of the frozen tissue (data not shown).

### Laser microdissection

**Fig. 8:**
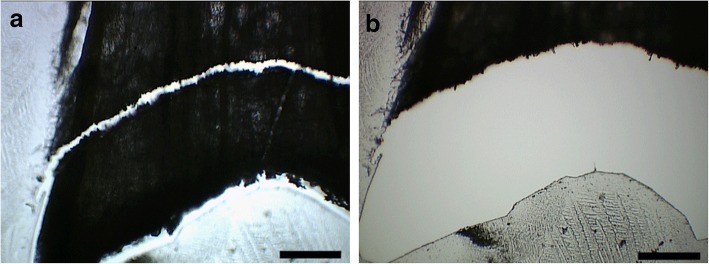
**a** Abscission zone selected to dissect from flower bud of poinsettia six days after decapitation (D6) on the laser microdissection microscope. **b** The same cryosection after laser microdissection. Bars 310 μm

4. The laser was able to dissect all thicknesses tested (data not shown). Nevertheless, the thickness of the cryosections at 300 and 350 μm made it more difficult to obtain the correct visualization of the abscission zone because the number of cell layers masked the three-dimensional shape. Thus, for our purpose, it was decided to use 250 μm (Fig. 8).
5. The area harvested using LMD for the average of the D0 samples (three replicates) was 61.8 ± 3.32 mm^2^, corresponding to 15.4 ± 0.83 mg of fresh tissue, while the average area for D6 (three replicates) was 54.9 ± 2.17 mm^2^, equivalent to 13.7 ± 0.53 mg of fresh weight (Table 1).

**Table 1:** Summary of collected average areas, fresh weight and auxin concentration in each replicate with standard errors of the mean from day 0 (D0) and six days after decapitation (D6) of abscission zones in poinsettia buds

	Sample name	Area mm^2^	Weight mg	Auxin ng/g FW
	D0-rep1	68.1	17	2.2
	D0-rep2	56.8	14.2	1.9
	D0-rep3	60.5	15.1	3.9
Average	D0	61.8 ± 1.92	15.4 ± 0.48	2.68 ± 0.62
	D6-rep1	55.7	13.9	2.9
	D6-rep2	58.2	14.6	4.9
	D6-rep3	50.8	12.7	2.2
Average	D6	54.9 ± 1.25	13.7 ± 0.31	3.34 ± 0.81

### Auxin quantification

**Fig. 9:**
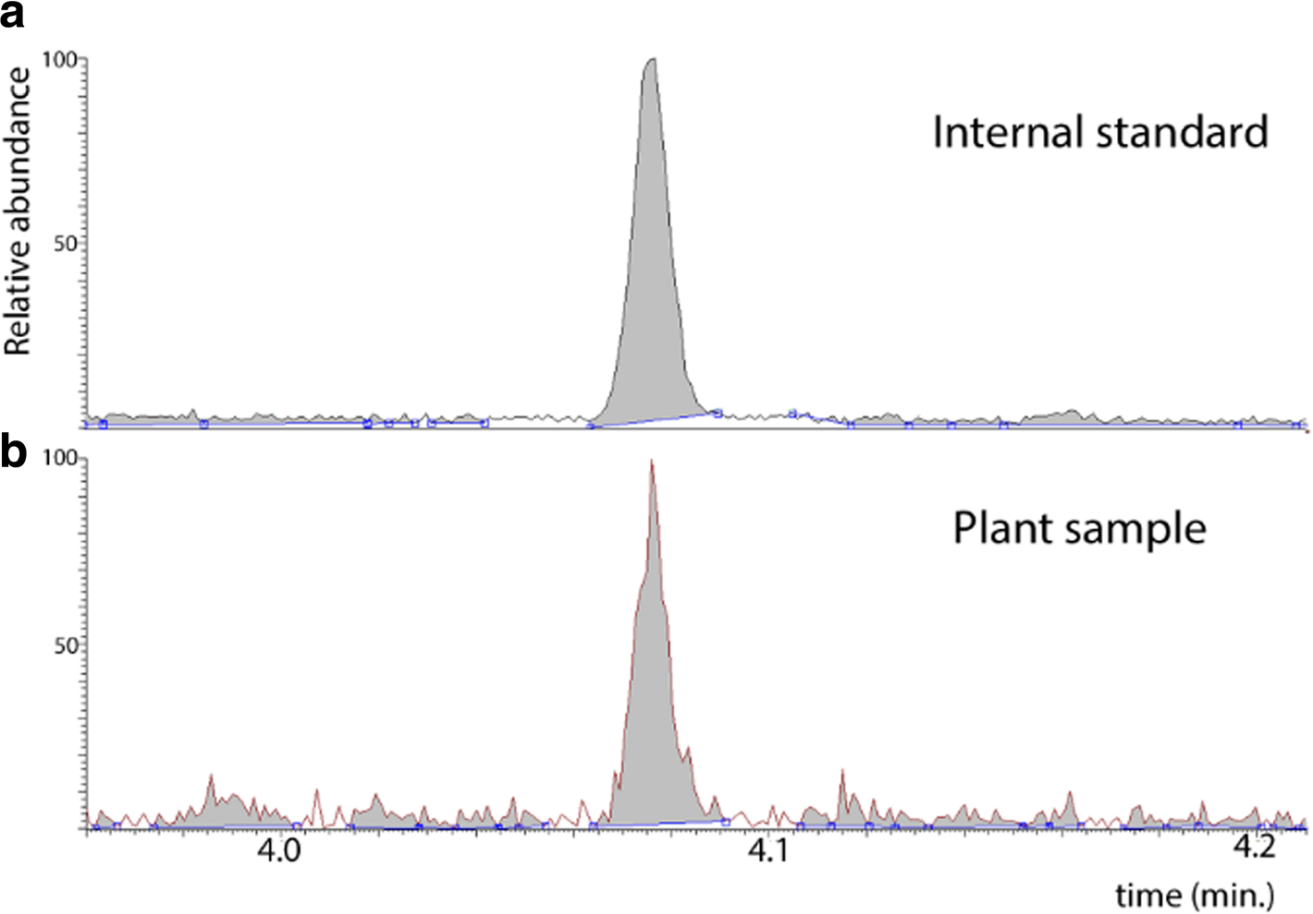
Chromatograph of auxin quantification in a poinsettia bud from laser microdissection microscope sampling combined with GC-SRM-MS for auxin analysis. **a** The internal standard [^13^C_6_]IAA. **b** Poinsettias bud sample corresponding to the abscission zone (AZ) from the day of decapitation (D0), i.e. a Control sample

6. The chromatogram peaks coincide with the internal standard, indicating the correct auxin identification (Fig. 9).
7. Auxin quantification using GC-MS/MS with cryosectioned, freeze-dried and microdissected tissue at different times of abscission zone development showed similar levels of endogenous auxin. For example, D0 and D6 auxin levels were 2.68 ± 0.63 ng/g FW and 3.34 ± 0.82 ng/g FW, respectively (Table 1).
8. Auxin quantification with validation samples of frozen cross sections containing abscission zones showed 19.92 ± 8.7 ng/g FW in D0 and 3.33 ± 0.29 ng/g FW in D6. These results confirm that IAA levels from the samples harvested with LMD fall into the same order of magnitude as the cross sections harvested in the control protocol using the cryostat.

## Discussion

Auxin quantification protocols for minute plant tissues are under constant development as procedures and equipment improve. The procedure used here was developed by us and first reported in 2012 [8]. It remains as one that needs a relatively small amount of tissue for a single assay, primarily because of the specificity provided by the selecting reaction monitoring (SRM) mode of the GC/MS-MS and precision is provided by the use of isotope dilution with a [^13^C]-labelled internal standard [20]. An important next step for improvement in auxin analysis is the ability to target with specificity the tissue of interest. However, even with the micro methods, the issues of IAA degradation during sample collection, determination of the amounts of tissue for these very small samples, and also how to collect enough of a specific tissue to obtain a good response in the MS remained significant problems. LMD emerged as a feasible way to select and harvest specific areas or cells of known thickness and surface area, but the pre-treatment of the sections, fixation and staining processes most commonly employed lead to the degradation and/or solubilisation of the hormones and other small molecules present in the tissue. This issue was largely overcome by cryosectioning the tissue, thus avoiding any further fixation and staining. An important additional step of freeze-drying with the help of a frozen aluminium block underneath the slide containing the whole cryosection contributed to the success of this protocol. Two factors were extremely important when deciding which conditions were optimal for treating the plant material: (1) minimizing the preparation time of the tissue to reduce the potential degradation of auxin and (2) obtaining enough material to get reliable quantification by mass spectrometry. Three parameters were evaluated: (1) the thickness of the cryosections, (2) the distinguishability of the abscission zone under the microscope, i.e. the ability to distinguish the macro structures we were targeting, and (3) the feasibility of using a laser to dissect these cryosections. The use of cryosectioning for plant tissues is not a common choice because the presence of vacuoles and cell walls makes it more difficult than for mammalian tissue samples [22]. Also, when they are used, it is very uncommon to find examples of cryosections with a thickness greater than 30 μm due to limitations of microscopy visualization (light penetration). This is especially true for some microscopy applications that require thin sections around 10 to 20 μm. However, as long as the structures remained easy to identify under the microscope, increasing the thickness proved to be beneficial in this case because it allowed us to collect larger amounts of tissue in a shorter time. When the downstream analysis involves low level metabolites or other barely traceable compounds, this can be a great advantage over, for example, protracted pre-treatment protocols or other methods.

## Conclusions

This is the first report of auxin quantification using microdissected plant materials harvested with LMD. Compared with some other plant hormones, auxin is present in relatively low concentration, is difficult to recover quantitatively, and its positional relocation via polar directed transport makes information on its spatial and temporal levels critical analytical challenges. Therefore, the importance of a reliable method for sample preparation, capable of providing tissue-specificity, is very significant and important. This protocol allows new approaches that will increase our knowledge concerning auxin distribution with spatial specificity within plant tissues. Since the sample preparation used in this study allowed us to exclude the use of solvents, the probability of auxin degradation was minimized. Thus, this protocol offers a clear advantage for exploring auxin concentrations at a more precise level of resolution and in a more straightforward manner. The protocol is suited for other applications such as transcriptomic, proteomics, metabolomics etc., where a high resolution in tissue harvesting is needed and minimal degradation of compounds is crucial for reliable results.

## Abbreviations

AZ: Abscission zone
FW: Fresh weight
GC-MS/MS: Gas chromatography coupled to tandem mass spectrometry
IAA: Indole-3-acetic-acid
LMD: Laser microdissection
OCT: Optimal cutting temperature
